# Supplementary material for: Replication study on the role of dopamine-dependent prefrontal reactivations in human extinction memory retrieval
Source: Nat Commun. 2024 Mar 27;15:2699. doi: 10.1038/s41467-024-46936-y (PMC10973457; doi:10.1038/s41467-024-46936-y)
Supplement: Supplementary file 3 — Reporting Summary [file 41467_2024_46936_MOESM3_ESM.pdf]

nature portfolio

Corresponding author(s): Elena Andres

Last updated by author(s): Nov 8, 2023

Reporting Summary

Nature Portfolio wishes to improve the reproducibility of the work that we publish. This form provides structure for consistency and transparency in reporting. For further information on Nature Portfolio policies, see our Editorial Policies and the Editorial Policy Checklist.

Statistics

For all statistical analyses, confirm that the following items are present in the figure legend, table legend, main text, or Methods section.

n/a	Confirmed	
☐	☒	The exact sample size (*n*) for each experimental group/condition, given as a discrete number and unit of measurement
☐	☒	A statement on whether measurements were taken from distinct samples or whether the same sample was measured repeatedly
☐	☒	The statistical test(s) used AND whether they are one- or two-sided<br>*Only common tests should be described solely by name; describe more complex techniques in the Methods section.*
☐	☒	A description of all covariates tested
☐	☒	A description of any assumptions or corrections, such as tests of normality and adjustment for multiple comparisons
☐	☒	A full description of the statistical parameters including central tendency (e.g. means) or other basic estimates (e.g. regression coefficient) AND variation (e.g. standard deviation) or associated estimates of uncertainty (e.g. confidence intervals)
☐	☒	For null hypothesis testing, the test statistic (e.g. *F*, *t*, *r*) with confidence intervals, effect sizes, degrees of freedom and *P* value noted<br>*Give P values as exact values whenever suitable.*
☒	☐	For Bayesian analysis, information on the choice of priors and Markov chain Monte Carlo settings
☒	☐	For hierarchical and complex designs, identification of the appropriate level for tests and full reporting of outcomes
☐	☒	Estimates of effect sizes (e.g. Cohen's *d*, Pearson's *r*), indicating how they were calculated

Our web collection on statistics for biologists contains articles on many of the points above.

Software and code

Policy information about availability of computer code

Data collection	We acquired the fMRI data using a 3Siemens MAGNETOM Trio 3 Tesla MRI System scanner. Visual stimuli were presented using Presentation Software (Presentation®, Neurobehavioral Systems, Inc., Berkeley, CA, USA).
Data analysis	Pre-processing and analysis of the fMRI data was performed using SPM12 (Wellcome Trust Centre for Neuroimaging, London, UK, http://www.fil.ion.ucl.ac.uk/) running on Matlab 2015b (MathWorks®, Natick, Massachusetts, USA). All statistical analyses of behavioral and psychophysiological data were implemented in R version 4.1.2 (2021-11-01). All analyses have been conducted using the following packages: ez (ANOVAs), car, MASS (regression analyses and diagnostics), pequod, emmeans (simple slope analyses), rstatix (statistical tests), irr (ICC), lmerTest (linear mixed effects models), coefplot, ggeffects, sjPlot stats, DescTools (diagnostics). The custom-made analysis script for scoring SCR running on Matlab 2015b as well as an R script for testing the main results can be accessed via https://doi.org/10.5281/zenodo.8353755.

For manuscripts utilizing custom algorithms or software that are central to the research but not yet described in published literature, software must be made available to editors and reviewers. We strongly encourage code deposition in a community repository (e.g. GitHub). See the Nature Portfolio guidelines for submitting code & software for further information.

Data

Policy information about availability of data

All manuscripts must include a data availability statement. This statement should provide the following information, where applicable:

- Accession codes, unique identifiers, or web links for publicly available datasets
- A description of any restrictions on data availability
- For clinical datasets or third party data, please ensure that the statement adheres to our policy

The sAA, sCORT, US expectancy, questionnaire and rating raw data generated in this study have been deposited in Zenodo under accession code https://doi.org/10.5281/zenodo.8353755. The raw MRI data are protected and are not available due to data privacy laws. The processed MRI data (vmPFC reactivations) and derived SCR data are available at the link provided above.
Further database used: Harvard-Oxford Atlas (Harvard Center for Morphometric Analysis)

Research involving human participants, their data, or biological material

Policy information about studies with human participants or human data. See also policy information about sex, gender (identity/presentation), and sexual orientation and race, ethnicity and racism.

Reporting on sex and gender	Seventy participants identifying themselves as male sex were included in the experiment based on the recruitment criteria, which explicitly sought male participants.
Reporting on race, ethnicity, or other socially relevant groupings	There were no socially constructed or socially relevant categorization variables used in the manuscript.
Population characteristics	All participants had normal or corrected-to-normal vision and reported no history of psychiatric, neurological or major medical problems. The age range was 25-39.
Recruitment	All participants were recruited through advertisements at the Univeristy Medical Campus, the University Campus, and other advertisement areas in the city of Mainz. Further, participants were recruited by online advertisements. Participants below the age of 25 or above the age of 39 were excluded. All participants gave informed consent. After experiment completion, participants were reimbursed 120 Euros. Participant selection bias was a potential concern, as there might have been an overrepresentation of medical or psychology students due to advertisements on the (medical) campus and their enhanced interest or motivation. This could have potentially led to faster learning rates if these individuals were more familiar with underlying processes. To mitigate this bias, we implemented the following precautions: participants who had previously taken part in a fear conditioning experiment in our facilities were excluded, and we expanded our advertising efforts beyond the campus, including online promotion. A post-hoc examination confirmed that the study/work backgrounds of participants were well-diversified, with only a marginal number being medical or psychology students. As a result, we consider the potential bias to be statistically insignificant.
Ethics oversight	The experiment was approved by the local ethics committee (Ethikkommission der Landesärztekammer, Rhineland-Palatinate, Germany) and was conducted in accordance with the Declaration of Helsinki.

Note that full information on the approval of the study protocol must also be provided in the manuscript.

Field-specific reporting

Please select the one below that is the best fit for your research. If you are not sure, read the appropriate sections before making your selection.

☒ Life sciences ☐ Behavioural & social sciences ☐ Ecological, evolutionary & environmental sciences

For a reference copy of the document with all sections, see nature.com/documents/nr-reporting-summary-flat.pdf

Life sciences study design

All studies must disclose on these points even when the disclosure is negative.

Sample size	To determine the optimal replication sample size, we combined different approaches. We conducted a power analysis based on the critical effect size from Gerlicher et al. (2018) for the most important hypothesis. This led to a required sample size of N=22 (i.e., eleven participants per treatment group). However, considering that effects in discovery samples may overestimate the true effect size, Simonsohn (2015) suggested that replication studies should have a sample size that is at least 2.5 times greater than that of the discovery study (N=40 in Gerlicher et al., 2018), leading to a required sample size of N=100 (50 per group). This approach would mean that we would need a sample size 4.5 times bigger than the sample size estimated based on the critical effect size in the discovery study, which we considered exaggerated. To balance the feasibility of a three-day pharmacological fMRI paradigm and the requirement to limit the number of participants exposed to study-associated burden with the requirement of sufficient statistical power, we settled on a sample size of N=70 (35 per group). With an estimated drop-out rate of 10%, this means we could expect to achieve a final sample size of N=63, which is 1.5 times greater than in the discovery study and nearly 3 times greater than based on power calculation.
Data exclusions	Skin conductance responses (SCRs): If more than 75% of trials during an experimental session were scored as zero, data of this participant

Data exclusions	during that session was regarded as invalid and excluded from SCR analysis, replicating the exclusion procedure in the discovery study (Gerlicher et al., 2018). Salivary alpha-amylase and cortisol data: No data was excluded, missing data (insufficient saliva for measurements) are indicated. MRI data: Data of participants was excluded when movement peaks exceeded more than 3 mm or 2° (task data, n=7, resting-state data: n=6 further participants).
Replication	The present study aimed to replicate the main study results of Gerlicher et al. (2018). We confirmed one of our three main hypotheses, whereas the results of our extensions to the study suggest a new possibility to explain vast individual differences and open up new avenues for further investigation. This study is the first replication attempt.
Randomization	Participants were randomly assigned to the L-DOPA or the placebo group using a randomization list generated before the start of the study, with the restriction that groups had to be matched on self-reported trait anxiety based on the State-Trait Anxiety Inventory questionnaire (STAI-T). STAI-T scores did not differ between groups after acquisition of n=45 participants, and therefore the predefined treatment group randomization order was kept.
Blinding	Drug preparation was done by a person not involved in the experiments or analyses. Investigators were blinded during data-collection and analysis.

Reporting for specific materials, systems and methods

We require information from authors about some types of materials, experimental systems and methods used in many studies. Here, indicate whether each material, system or method listed is relevant to your study. If you are not sure if a list item applies to your research, read the appropriate section before selecting a response.

Materials & experimental systems

n/a	Involved in the study
☒	☐ Antibodies
☒	☐ Eukaryotic cell lines
☒	☐ Palaeontology and archaeology
☒	☐ Animals and other organisms
☒	☐ Clinical data
☒	☐ Dual use research of concern
☒	☐ Plants

Methods

n/a	Involved in the study
☒	☐ ChIP-seq
☒	☐ Flow cytometry
☐	☒ MRI-based neuroimaging

Plants

Seed stocks	*Report on the source of all seed stocks or other plant material used. If applicable, state the seed stock centre and catalogue number. If plant specimens were collected from the field, describe the collection location, date and sampling procedures.*
Novel plant genotypes	*Describe the methods by which all novel plant genotypes were produced. This includes those generated by transgenic approaches, gene editing, chemical/radiation-based mutagenesis and hybridization. For transgenic lines, describe the transformation method, the number of independent lines analyzed and the generation upon which experiments were performed. For gene-edited lines, describe the editor used, the endogenous sequence targeted for editing, the targeting guide RNA sequence (if applicable) and how the editor was applied.*
Authentication	*Describe any authentication procedures for each seed stock used or novel genotype generated. Describe any experiments used to assess the effect of a mutation and, where applicable, how potential secondary effects (e.g. second site T-DNA insertions, mosiacism, off-target gene editing) were examined.*

Magnetic resonance imaging

Experimental design

Design type	Task (event related) and resting state.
Design specifications	Day 1: 20 trials; Day 2: 30 trials; Day 3: 40 trials; trial length: 4.5 s; ITI: 17, 18, or 19 s
Behavioral performance measures	US expectancy ratings before and after each experimental session (response rate 100%)

Acquisition

Imaging type(s)	functional, structural, diffusion
Field strength	3T
Sequence & imaging parameters	MRI data was acquired on a Siemens MAGNETOM Trio 3 Tesla MRI System using a 32-channel head coil. Resting-state and task data were recorded using gradient echo, echo planar imaging (EPI) with a multiband sequence covering the whole brain (TR: 1000 ms, TE: 29 ms, multi-band acceleration factor: 4, voxel-size: 2.5 mm isotropic, flip angle 56°, field

of view: 210 mm). A high-resolution T1 weighted image was acquired after the experiment on day 1 for anatomical visualization and normalization of the EPI data (TR: 1900 ms, TE: 2540 ms, voxel size: 0.8 mm isotropic, flip angle 9°, field of view: 260 mm, MPRAGE sequence). T2 weighted images were collected for preventative neuro-radiological diagnostics for all participants (45 slices, TR: 6100 ms, TE: 79 ms, voxel size: 3 mm isotropic, flip angle: 120°, Turbo Spin Echo (TSE) sequence). Lastly, we collected multidimensional diffusion-weighted images (DWI) from each participant (72 slices, voxel-size: 2 mm isotropic, TR: 9100 ms, TE: 85 ms, number of directions: 64, diffusion weights: 2, b-value 1: 0 s/mm2, b-value 2: 1000 s/mm2, Multi-Directional Diffusion Weighted (MDDW) sequence)). DWI were not further considered.

Area of acquisition: whole brain

Diffusion MRI: ☐ Used ☒ Not used

Preprocessing

Preprocessing software: fMRI data was preprocessed and analyzed using statistical parametric mapping (SPM12, Wellcome Trust Centre for Neuroimaging, London, UK, http://www.fil.ion.ucl.ac.uk/) running on Matlab 2015b (MathWorks®, Natick, Massachusetts, USA). Preprocessing included realignment and co-registration, normalization and smoothing using a 6mm full-width-at-half-maximum Gaussian smoothing kernel.

Normalization: The T1 weighted anatomical image was segmented and normalized to add Montreal Neurological Institute (MNI) space based on SPM's tissue probability maps. Normalization of the functional images was achieved by applying the resulting deformation fields to the realigned and co-registered functional images.

Normalization template: Standard space MNI brain provided in SPM12 (http://www.fil.ion.ucl.ac.uk)

Noise and artifact removal: Preprocessing includes realignment and co-registration of the mean functional image to the T1 weighted anatomical image. Functional data was smoothed using a 6mm full-width-at-half-maximum Gaussian smoothing kernel. Data of participants was excluded when movement peaks exceeded more than 3 mm or 2°.

Volume censoring: The first 5 volumes of each scan were discarded due to equilibrium effects.

Statistical modeling & inference

Model type and settings: Investigating potential reactivations of extinction specific multi-voxel patterns (MVPs) in the vmPFC, we analyzed the extinction task data (day 2) using a model including one regressor for CS+ and CS- onsets, respectively, US-expectancy ratings, and context on/-offset. Furthermore, the model included one regressor for the first five CS+ offsets, where omission of the US was unexpected, and one for the first five CS- offsets, where US omission was expected, as well as one regressor each for the remaining ten CS+ and CS- offsets. All regressors were delta-functions convolved with the hemodynamic response function (HRF). The MVP evoked by the first five US omissions at CS+ offset in the vmPFC was extracted from the resulting beta-map in the vmPFC region of interest. Resting-state data was analyzed in accordance with a previous study examining memory reactivation (Gerlicher et al., 2018), i.e., general linear models (GLMs) for each day 2 resting-state scan (pre-, ~10, 45 and 90 minutes post-extinction) included delta-function regressors for each volume (TR: 1 sec), thereby accounting for potential reactivations which may have occurred during any point of the resting-state scan. No high-pass filtering was applied in the resting-state models and AR(1) auto-correlation correction was employed. MVPs in the pre-defined ROI during the resting state were extracted from the resulting beta-image series (TR = 1 second, i.e., 480 – 5 = 475 beta images). Subsequently, we correlated (Pearson correlation coefficient) the pattern evoked by the first five US omissions at CS+ offset during extinction with the resulting 475 patterns of all four resting-state scans and Fisher Z- transformed the correlation coefficients.

Effect(s) tested: Multiple linear regression analyses with number of CS+ offset-related vmPFC MVP at baseline, ~10, 45, and 90 min after extinction as predictors and average differential (CS+>CS-) SCR during either the spontaneous recovery or the renewal test as dependent variables were performed separately.

Specify type of analysis: ☐ Whole brain ☒ ROI-based ☐ Both

Anatomical location(s): The 475 correlations of the MVP with the resting-state pattern recorded before extinction learning was employed to create a baseline distribution. The mean and standard deviation of this baseline distribution were used to transform each correlation between the MVP and the postextinction resting- state patterns into a Z-score ($Z_i = (r_i - \mu_i)/\sigma$). Correlations with a Z-score exceeding a value of 2 ($Z > 2 \approx p < .05$) were counted as potential reactivations of the CS+ offset-related vmPFC pattern. Reactivations were summed per participant and resting-state scan.

Statistic type for inference (See Eklund et al. 2016): Voxel-wise

Correction: The 475 correlations of the MVP with the resting-state pattern recorded before extinction learning was employed to create a baseline distribution. The mean and standard deviation of this baseline distribution were used to transform each correlation between the MVP and the postextinction resting- state patterns into a Z-score ($Z_i = (r_i - \mu_i)/\sigma$). Correlations with a Z-score exceeding a value of 2 ($Z > 2 \approx p < .05$) were counted as potential reactivations of the CS+ offset-related vmPFC pattern. Reactivations were summed per participant and resting-state scan.

Models & analysis

n/a	Involved in the study
☒	☐ Functional and/or effective connectivity
☒	☐ Graph analysis
☐	☒ Multivariate modeling or predictive analysis

Multivariate modeling and predictive analysis: see above
